# Type X collagen levels are elevated in serum from human osteoarthritis patients and associated with biomarkers of cartilage degradation and inflammation

**DOI:** 10.1186/1471-2474-15-309

**Published:** 2014-09-22

**Authors:** Yi He, Anne Sofie Siebuhr, Niels Ulrik Brandt-Hansen, Jianxia Wang, Di Su, Qinlong Zheng, Ole Simonsen, Kristian Kjær Petersen, Lars Arendt-Nielsen, Thomas Eskehave, Hans Christian Hoeck, Morten Asser Karsdal, Anne C Bay-Jensen

**Affiliations:** Nordic Bioscience, Herlev Hovedgade 207, DK-2730 Herlev, Denmark; Nordic Bioscience China, Zhongguancun Life Science Park, 102206 Beijing, P.R. China; Department of Orthopedic Surgery, Aalborg University Hospital, Hobrovej 19, 9000 Aalborg, Denmark; Department of Health Science and Technology, Center for Sensory-Motor Interaction, Aalborg University, Aalborg, Denmark; Center for Clinicl and Basic Research and C4Pain, Aalborg, Denmark

**Keywords:** Hypertrophic chondrocytes, Osteoarthritis, Type X collagen, Cartilage degradation, Biomarkers, Type II collagen, Inflammation

## Abstract

**Background:**

Osteoarthritis (OA) is the most common degenerative joint disease, of which the pathogenesis is inadequately understood. Hypertrophy-like changes have been observed as part of the progression of OA. The aim of the study was to develop and characterize a novel biomarker of chondrocytes hypertrophy and investigate how this marker was associated with cartilage degradation and inflammation in patients with various degrees of OA.

**Methods:**

A competitive ELISA, C-Col10, applying a well-characterized monoclonal antibody was developed as a biomarker of chondrocyte hypertrophy through measurement of type X collagen (ColX). The levels of C-Col10, C2M (matrix metalloproteinase-derived fragments of type II collagen) and hsCRP (high sensitive C-reactive protein) were quantified by ELISAs in serum of 271 OA patients stratified by Kellgren-Lawrence (KL) score 0–4. Associations between serum levels of the three biomarkers (log transformed) were analyzed by Pearson’s correlation and differences in C-Col10 levels between patients with high and low levels of inflammation measured by hsCRP were analyzed by ANOVA.

**Results:**

We developed a C-Col10 assay measuring the C-terminus of ColX. We found significantly higher levels of ColX in patients with KL score 2 compared to patients with no radiographic evidence of OA (KL0) (p = 0.04). Levels of ColX were significantly elevated in OA patients with above normal hsCRP levels (p < 0.0001), as well as significantly correlated with levels of C2M (r = 0.55, p < 0.0001), which suggested that chondrocyte hypertrophy was associated with inflammation and cartilage degradation. There was no correlation between C2M and hsCRP. Age and BMI adjustment didn’t change the results. Immuno-staining revealed that ColX was predominately located around the hypertrophic chondrocytes and the clustered chondrocytes indicating that C-Col10 measures may be linked to cartilage hypertrophic changes.

**Conclusions:**

We developed a novel assay, C-Col10, for measurement of chondrocyte hypertrophy and found its levels significantly elevated in OA patients with KL score of 2, and also in OA patients with above normal hsCRP levels. Concentration of C-Col10 strongly correlated with levels of C2M, a marker of cartilage destruction. The data suggest that chondrocyte hypertrophy and subsequent collagen X fragmentation seem to be increased in a subset of patients with inflammatory OA.

**Electronic supplementary material:**

The online version of this article (doi:10.1186/1471-2474-15-309) contains supplementary material, which is available to authorized users.

## Background

Osteoarthritis (OA) is the most common joint disease, which is characterized by cartilage damage and loss of joint function. The high prevalence, with the accompanying disabilities of OA results in a huge economic burden on society [1]. However, the molecular mechanism of OA disease remains partly understood and is more complicated than expected. Not only the changes in extracellular matrix (ECM) composition, but also changes in the metabolism of chondrocytes contribute to the progression of OA [2]. The events of chondrocytes hypertrophy differentiation including matrix degradation, neo-vessels invasion from subchondral bone and matrix calcification have been observed in the progression of OA, which mimic the processes during skeletal development by endochondral ossification [3–6]. Runt-related transcription factor 2 (RUNX2), a transcription regulator of type X collagen, has been reported to be involved in the development of OA disease [7]. The critical role of hypertrophy chondrocytes have been summarized in several good reviews [8–10].

Type X collagen (ColX) is a well-established marker for hypertrophic chondrocytes differentiation, which is a non-fibrillar collagen consisting of three identical alpha 1 chains. Each chain has three domains: a short triple helix domain flanked by a bigger globular domain (NC1 domain) at the carboxyl end and a short non-collagenous domain (NC2 domain) at the amino end [11]. The molecular weight of each alpha 1 chain is 64 kDa of human ColX, of which triple helix domain is 42 kDa [12]. ColX is susceptible to interstitial collagenase and gelatinase cleavage at two distinct sites within triple helix domain generating fragments [13]. ColX is considered to be synthesized specifically by hypertrophic chondrocytes of growth plate during the development of long bone [14]. Immunoelectron microscopy shows that individual ColX molecules can rapidly assemble into a hexagonal lattice via carboxy-terminal globular domains, which play the important role in modifying the cartilage matrix for the subsequent bone formation during endochondral ossification [15]. Besides the maintenance of tissue stiffness, several other roles have been suggested, including regulating chondrocytes metabolism and interacting with hypertrophic chondrocytes through cell adhesion molecules such as integrins [16]. There is increasing evidence that the expression of ColX is elevated in human OA cartilage as a result of chondrocytes hypertrophy and cartilage calcification [17–19]. The up-regulation of ColX has been reported in experimental animal OA models as well [20–23].

OA is thought to be a “non-inflammatory” joint disease due to low level of inflammation in contrast to other joint disease such as rheumatoid arthritis (RA). Despite that, inflammation has indeed been considered to contribute to the symptoms and progression of OA [24, 25]. Circulating markers of inflammation - such as C-reactive protein (CRP), a common diagnostic marker for chronic inflammatory disease such as RA - has been shown to be elevated in subsets of OA patients as compared to a sample population without disease [26]. Elevated serum CRP may reflect inflammation in affected joints, like synovitis, which is associated with clinical symptoms such as pain [27]. It was reported by Cecil et al. that inflammation-induced chondrocytes hypertrophy is contributed significantly to the progression of OA [28]. Thus, it is interesting to investigate the relationship between hypertrophy and inflammation in OA.

The aim of this study was to develop an immunoassay (C-Col10) to determine the levels of ColX in blood and to investigate the relationships between chondrocyte hypertrophy, cartilage degradation and systemic inflammation by measuring three biomarkers in serum from 271 OA patients: C-Col10, C2M and high sensitive (hs) CRP. Serum C2M measures circulating fragments of type II collagen, a surrogate marker of cartilage degradation. C2M was found to be significantly higher in patients with moderate/severe OA compared to patients with mild OA or healthy age-matched controls [29], as well as in patients with ankylosing spondylitis (AS) compared to controls [30].

## Methods

### Materials

Materials and chemicals were purchased from Sigma-Aldrich (Broendby, Denmark) or VWR (Roedovre, Denmark) if not stated otherwise.

### Serum samples from OA patients

Serum samples were retrieved from C4Pain study (n = 271 with Kellgren-Lawrence score ranging from 0–4). In this study, the OA population was recruited based on intensity of knee joint pain, ranging from 0 to 100 on a Western Ontario and McMaster Universities Osteoarthritis Index (WOMAC) pain scale. Two plain X-ray examinations in standing position were performed. Serum was collected upon overnight fasting prior to surgery or during consultation. The study was approved by The Ethical Committee of Northern Jutland (VEK no.: N-20100094). It was conducted according to the Principal of Good Clinical Practice and according to the Declaration of Helsinki. All patients provided written informed consent.

### Development and characterization of anti-ColX monoclonal antibody

A specific peptide, SFSGFLVAPM obtained from C-terminus of NC1 domain of ColX was synthesized and conjugated to maleimide-activated keyhole-limpet hemocyanin (KLH, Pierce, Beijing, China) as immunogen. The immunogens were used to immunize female, seven-week-old Balb/C mice by repeating injections. The monoclonal antibody was produced by standard method. The monoclonal antibody was screened and characterized by competitive ELISA by using the specific peptide (SFSGFLVAPM), the truncated peptide (SFSGFLVA) without last 2 amino acids and the non-sense peptide (DMDYLPRVPNQ).

### Western Blotting of U2-OS cell lysates

Further characterization of ColX monoclonal antibody, NB509-11G8, was conducted by western blotting with cell lysates of human osteosarcoma cell line, U2-OS expressing type X collagen. The cell lysates were prepared using fresh RIPA buffer (25 mM Tris–HCl pH7.6, 150 mM NaCl, 1 Sodium deoxycholate acid and fresh EDTA-free protease inhibitor cocktail tablet (Roche, USA)). U2-OS lysates were separated in 4–12 Bis-Tris gradient gel and electrically transferred to polyvinylidene fluoride (PVDF) membrane. After blocking, the membrane was incubated with 1 μg/ml NB509-11G8 antibody or X53 (positive control for intact ColX) at 4°C overnight. To confirm the specificity of bands, the peptide inhibition western blotting was performed in parallel with adding 3 μg/ml selection peptide or truncated peptide into NB509-11G8 antibody solution, then immediately incubated with the membrane. After incubation with goat anti-mouse secondary antibody (1:5000) at RT (room temperature) for 2 hr, the membrane was washed and detected using enhanced chemiluminescence (ECL) western blotting substrate (GE healthcare, Denmark). The bands were visualized through exposure to X-ray film.

To investigate the possibility of NB509-11G8 detecting type X collagen fragments, the *in vitro* digestion of U2-OS lysates was carried out by degradation of collagenase (C6885, Sigma) with two concentrations, 50 μg/ml and 5 μg/ml. The cleavage reactions were carried out for 1 hr, 2 hr, 4 hr or overnight (20 ± 1 hr) at 37°C. These mixtures were submitted to western blotting and detected by NB509-11G8 antibody and X53.

### Western blotting of human OA cartilage extracts

Cartilage biopsies were obtained from 3 OA patients who underwent total knee arthroplasty. Proteins were extracted with 1 ml 4 M guanidinium chloride (GuHCl) containing 50 mM Sodium Acetate, 10 mM EDTA, 0.1 M Hexanoic Acid, pH5.8 at 4°C for 48 hr. The extract was separated from cartilage residue by centrifugation (800 g) at 4°C for 10 min and stored at −70°C prior to use. Inhibition western blotting as mentioned above was applied to detect type X collagen in three human OA cartilage extracts (human skeletal muscle extract used as negative control from Biochain, USA).

### Development and characterization of the C-Col10 ELISA

The competitive C-Col10 ELISA was developed with optimal mix of buffer, incubation time, temperature and concentrations of reagents. The final protocol was as follows: 100 μl biotinylated peptide was added to a streptavidin pre-coated plate and incubated at 20°C for 30 min. Next, the plate was washed 5 times with standard wash buffer. 20 μl standards or samples together with 100 μl peroxidase labeled antibody were added to the plate and incubated at 4°C overnight with shaking. After that, the wells were washed 5 times and 100 μl/well 3,3’,5,5’-tetramethylbenzidine (TMB) was added and incubated in the dark at 20°C for 15 min. Lastly, 100 μl/well stopping solution (0.1 H_2_SO_4_) was added and the colorimetric reaction was measured at 450 nm with reference at 650 nm. Technical assay validation was done according to the international guide.

### Biochemical markers

ColX, cartilage degradation and systemic inflammation were quantified in serum of 271 OA patients by 3 assays: C-Col10, C2M and hsCRP (Siemens 74701). C-Col10 assay was followed the protocol above mentioned, while hsCRP assay was strictly followed the protocol recommended in the kit manual. C2M ELISA assay was followed the protocol described previously, which has been used in several studies [30, 31].

### *In situ* detection of ColX in human OA cartilage

Cartilage biopsies from 3 OA patients were taken from OA patients undergoing total knee replacement surgery at the Department of Orthopedics at Sygehus Vendsyssel, Frederikshavn, Denmark. The retrieval of specimens complied with international ethical guidelines for handling human samples and patient information. All participants signed an informed consent form and the study was approved by Danish National Ethical Committees under the Act on Research Ethics Review of Health Research Projects (journal no. N-20110031).

The cartilage biopsies were fixed in formaldehyde, decalcified with EDTA, and embedded in paraffin. Sections were cut into 5 μm adjacent sections and melted at 60°C, deparaffinized and hydrated. Endogenous Peroxidase activity was blocked with H_2_O_2_ in 99 ethanol by incubation at RT for 20 min. For C-Col10, antigen retrieval was done by Pronase E (Roche) at 37°C for 15 min. For C2M, citrate buffer pH6.0 at 60°C overnight was used to unmask antigen. Unspecific protein binding was blocked with 0.5 casein in TBS buffer at RT for 30 min. Afterward, NB509- 11G8 antibody, the C2M antibody or normal mouse IgG (negative control) were incubated with sections at 4°C overnight. Immunoactivity was detected by using peroxidase labeled secondary antibody and diminobenzidine (DAB, Dako, Denmark) as chromogen. Sections were counter stained with Mayer’s acidic hematoxylin for 12 sec. Pictures were taken with a digital camera (Olympus C5050) on a microscope (Olympus BX60).

### Statistics

Statistic analysis of correlation was performed using GraphPad Prism^@^ version 5. Levels of the 3 biomarkers in serum samples were shown as mean [95-CI]. One-way ANOVA was applied when compare biomarker levels. Correlations between levels of biomarkers were done by Pearson’s test on log transformed data.

## Results

### The specificity of the monoclonal antibody NB509-11G8

**Figure 1:**
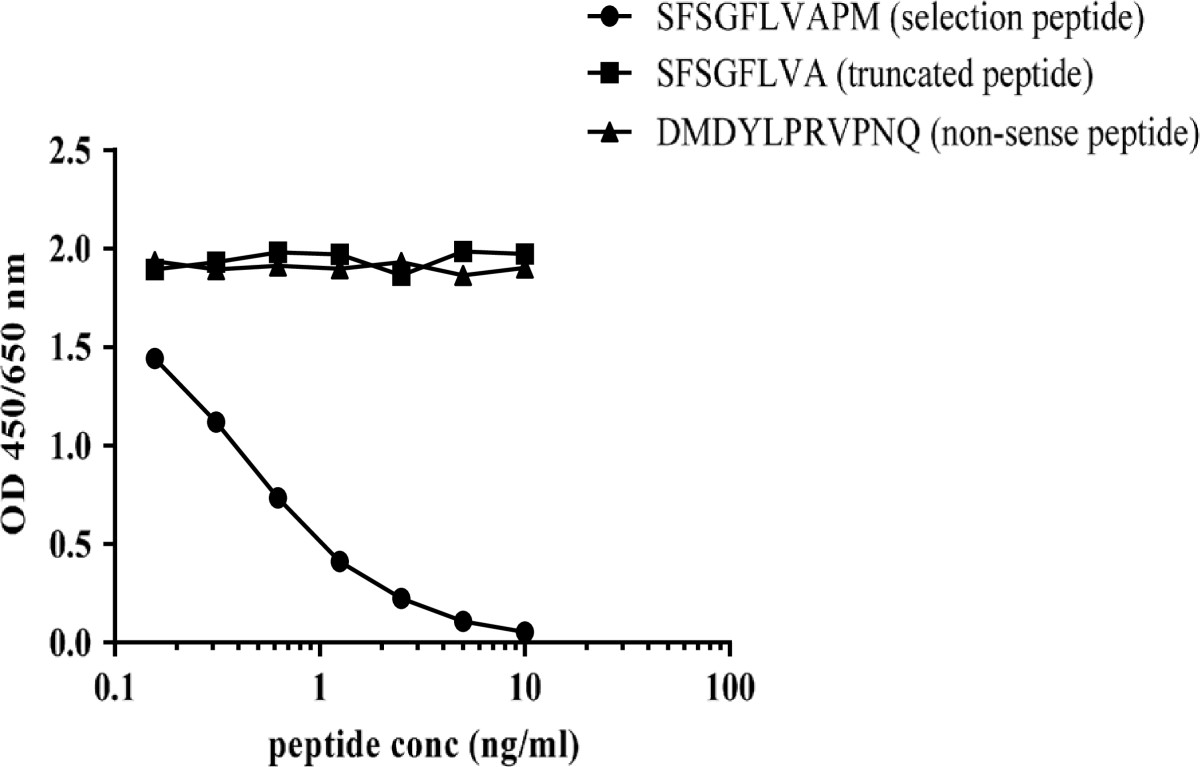
**Peptide specificity test.** The peptide specificity was evaluated by three synthetic peptides in inhibition ELISA: the selection peptide (SFSGFLVAPM), the truncated peptide (SFSGFLVM) and the non**-** sense peptide (DMDYLPRVPNQ). The OD signal was dramatically inhibited by the selection peptide, but not the truncated peptide nor the non-sense peptide.

The sequence alignment of species is shown in Table 1.The signal of NB509-11G8 (IgG1, κ) was displaced by increasing concentration of selection peptide (SFSGFLVAPM), but neither by the truncated peptide (SFSGFLVA) nor by the non-sense peptide (DMDYLPRVPNQ) (Figure 1), indicating that NB509-11G8 specifically recognized the unique C-terminus of NC1 domain.

**Table 1:** **Sequence alignment of the last 10 aa of NC**-**1 domain of ColX α1 chain in different species**

Species	Sequence	Database
Homo sapiens^1^	SFSGFLVAPM	GenBank:CAA46236.1
Mus musculus^2^	SFSGFLVAPM	GenBank:CAA46237.1
Bos taurus^3^	SFSGFLVAPM	NCBI: NP_777059.1
Rattus norvegicus^4^	SFSGFLVAP *I*	GenBank: CAA10518.1
Canis lupus familiaris^5^	SFSGFLVAPM	NCBI: XP_003639449.1
Equus caballus^6^	SFSGFLVAPM	NCBI: XP_001504151.1

^1^Human; ^2^Mouse; ^3^Cattle; ^4^Rat; ^5^Dog; ^6^Horse.

### Detection of ColX by the monoclonal antibody NB509-11G8 in U2-OS cell lysates and human cartilage extracts

**Figure 2:**
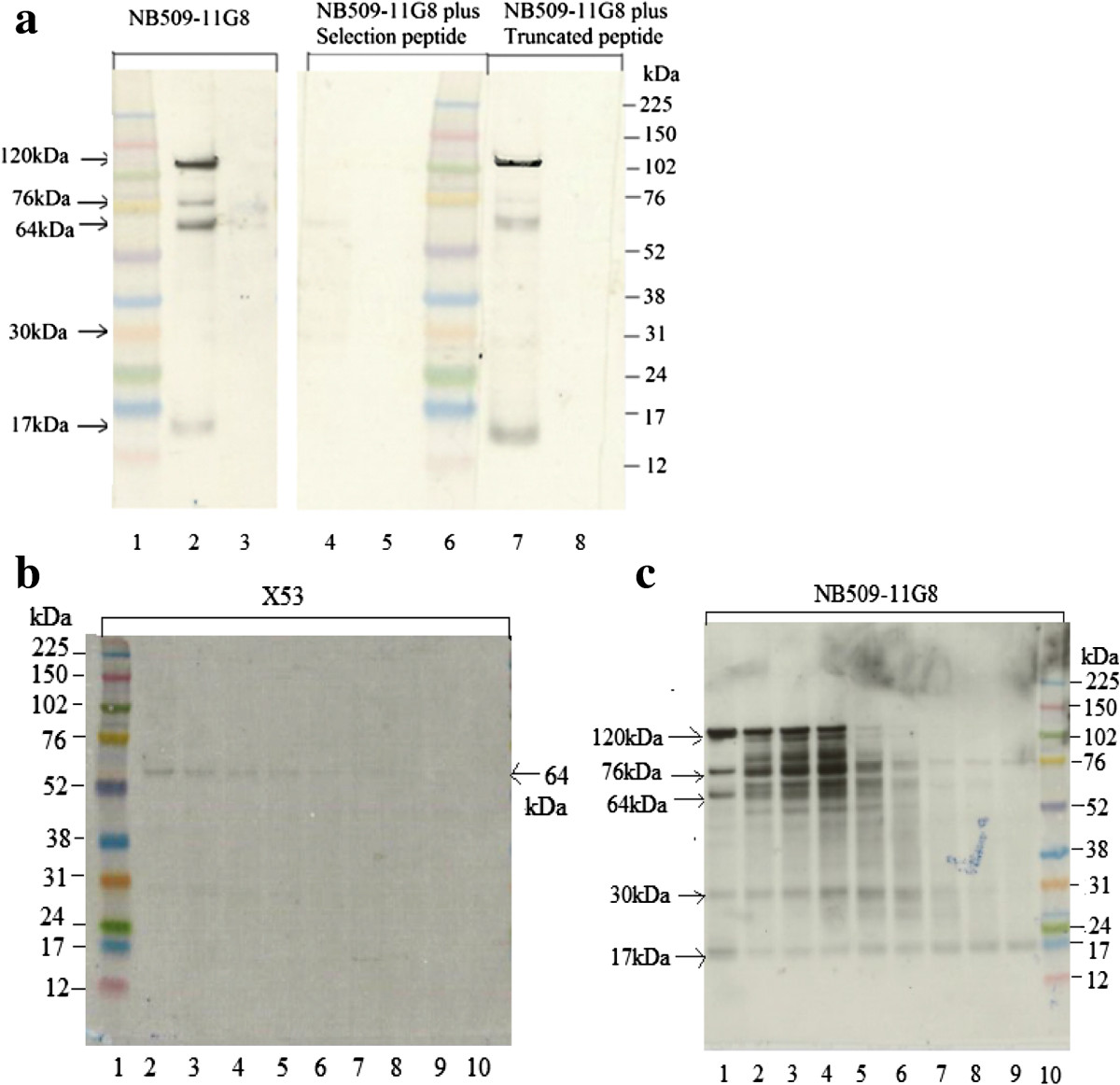
**Western blotting on U2**-**OS cell lysates. A**. Lane 1 and 6: marker. Lane 2, 4 and 7: U2-OS cell lysates; Lane 3, 5 and 8: RIPA buffer. 5 bands have been identified in U2-OS cell lysates by NB509-11G8. Moreover, these bands can be completely blocked by the selection peptide, but not the truncated peptide. **B**. U2-OS cell lysates in vitro digestion products detected by X53 on western blotting. Lane1: marker. Lane 2: U2-OS cell lysates; Lane 3–6: U2-OS cell lysates incubated with 5 ug/ml collagenase for 1 hr, 2 hr, 4 hr or overnight. Lane7-10: U2-OS cell lysates incubated with 50 ug/ml collagenase for 1 hr, 2 hr, 4 hr or overnight. X53 only detected 64 kDa α1 chain. The intensity of this chain decreased with the incubation time and the amount of collagenase. **C**. U2-OS cell lysates in vitro digestion products detected by NB509-11G8 on western blotting. Lane 1: U2-OS cell lysates; Lane 2–5: U2-OS cell lysates incubated with 5 ug/ml collagenase for 1 hr, 2 hr, 4 hr and overnight. Lane6-9: U2-OS cell lysates incubated with 50 ug/ml collagenase for 1 hr, 2 hr, 4 hr or overnight. Lane10: marker. The degradation of ColX by collagenase exhibited a time- and dose- dependent pattern.

**Figure 3:**
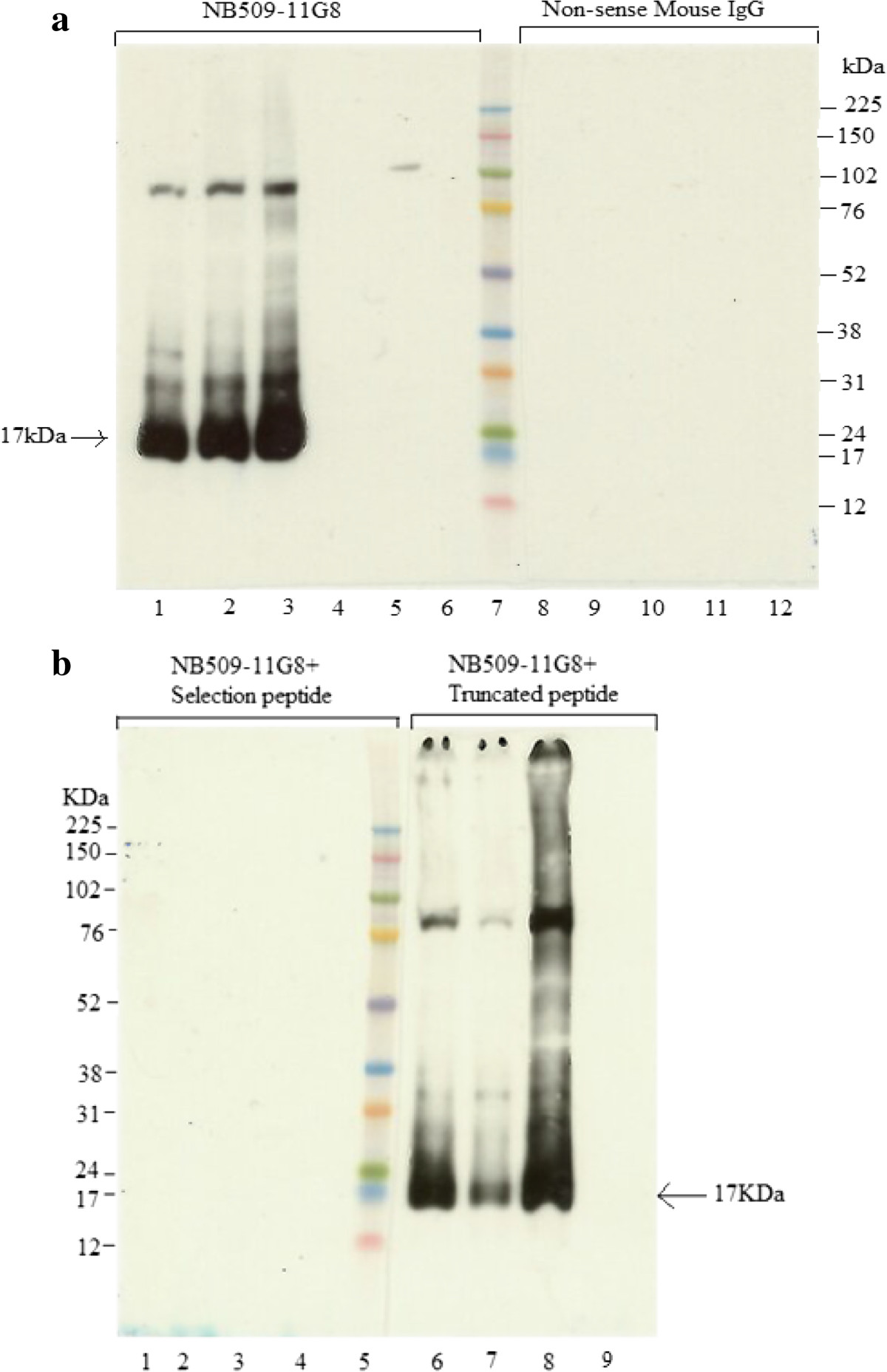
**Western blotting on human OA cartilage extract. A**. Lane1 and 8: human OA cartilage extract1; Lane 2 and 9: human OA cartilage extract 2; Lane 3 and 10: human OA cartilage extract3; Lane 4 and 11: GuHCl extraction buffer. Lane 5 and 12: human skeletal muscle extract; Lane 6: muscle extraction buffer. Lane 7: marker. Lane 1–7 was incubated with NB509-11G8, while Lane 8–12 was incubated with non-sense mouse IgG. The same size of 17 kDa band was visualized in three cartilage extract, but not in the muscle extract. No any band was detected in neither cartilage extract nor muscle extract by non-sense mouse IgG. **B**. Peptide inhibition western blotting on human OA cartilage extracts. Lane 5: marker; Lane 1and 6: human OA cartilage extract1; Lane 2 and 7: human OA cartilage extract2; Lane 3 and 8: human OA cartilage extract3. Lane 4 and 9: GuHCl extraction buffer. The 17 kDa bands in 3 human OA extracts were blocked by selected peptide, but not truncated peptide.

U2-OS is a widely used human osteo/chondroblast-like cell line derived from malignant bone tumors with the capacity to express ColX [32]. Commercial X53 antibody recognized only intact α1 chain (Figure 2B, lane 2), whereas NB509-11G8 detected five bands (Figure 2A): i) a 17 kDa band, corresponding to a previously reported band generated by intestinal collagenase (MMP-1) [13]; ii) a 30 kDa band with a unknown cleavage site in triple helix domain; iii) a 64 kDa band representing the intact α1 strand; iv) an unknown 76 kDa band, and v) an unknown 120 kDa band. The difference in bands detected by X53 and NB509-11G8 might be explained by the fact that they target against different sequences. The epitope of X53 has been suggested to recognize a pepsin-resistant epitope overlapping collagenous domain and portion of NC-1 domain. All bands were absent, when the selection peptide was added into NB509-11G8 solution, indicating that all bands carry the C-terminus of NC-1 domain.The degradation of ColX by collagenase showed a time- and dose- dependent pattern, which was demonstrated using both X53 and NB509-11G8 (Figure 2B and 2C). The α1 chain following digestion exhibited greatly reduced intensity when stained by X53 (Figure 2B). Digestion with 5 μg/ml collagenase released more fragments detected by NB509-11G8(Figure 2C, lane 2–4) than before treatment (lane 1). The 120 kDa band became weaker over incubation time and completely disappeared after overnight incubation, whereas a 32 kDa band was more clearly visible. Meanwhile, 50 μg/ml collagenase aggressively degraded ColX (lane 6–9). The 17 kDa band were unaffected by the collagenase treatment.A major 17 kDa-fragment was identified in three different human OA cartilage extracts (Figure 3A), but not in human skeletal muscle extract. No any band was detected by the non-sense mouse IgG (negative control). Moreover, the bands were completely blocked by the selection peptide instead of the truncated peptide, demonstrating the 17 kDa fragment contains the C-terminus of ColX (Figure 3B).

### The technical performance of the C-Col10 ELISA

A competitive ELISA was developed by applying the NB509-11G8 monoclonal antibody. The technical performance of this ELISA is summarized in Table 2. The LLOD was 24 pg/ml. The intra-assay CV was 4.19 and the inter-assay CV was 13.2. The measurement range was 40–7300 pg/ml and the IC50 was 363 pg/ml. The dilution recovery and peptide spiking recovery test in human serum were 100 ± 20 within the measurement rang of the assay.

**Table 2:** **Summary of technical performance for 3 biomarkers assays**

	C-Col10 (pg/ml)	hsCRP (μg/ml)	C2M (pg/ml)
***Assay specifications***			
Slope of standard curve	1.23	n. a.	0.96
IC50, pg/ml	363	n. a.	440
Intra-assay CV%	4.19	n. a.	3.95
Inter-assay CV%	13.18	<5	9.88
Lower limit of detection	24	n. a.	n. a.
Quantifiable range	40-7300	0.2-10	30-1700

C-Col10: C-terminus of ColX assay; C2M: MMP-derived collagen type II fragment assay; hsCRP: high sensitive CRP assay. n.a.: not available.

### Biomarker levels in the C4Pain cohort

**Figure 4:**
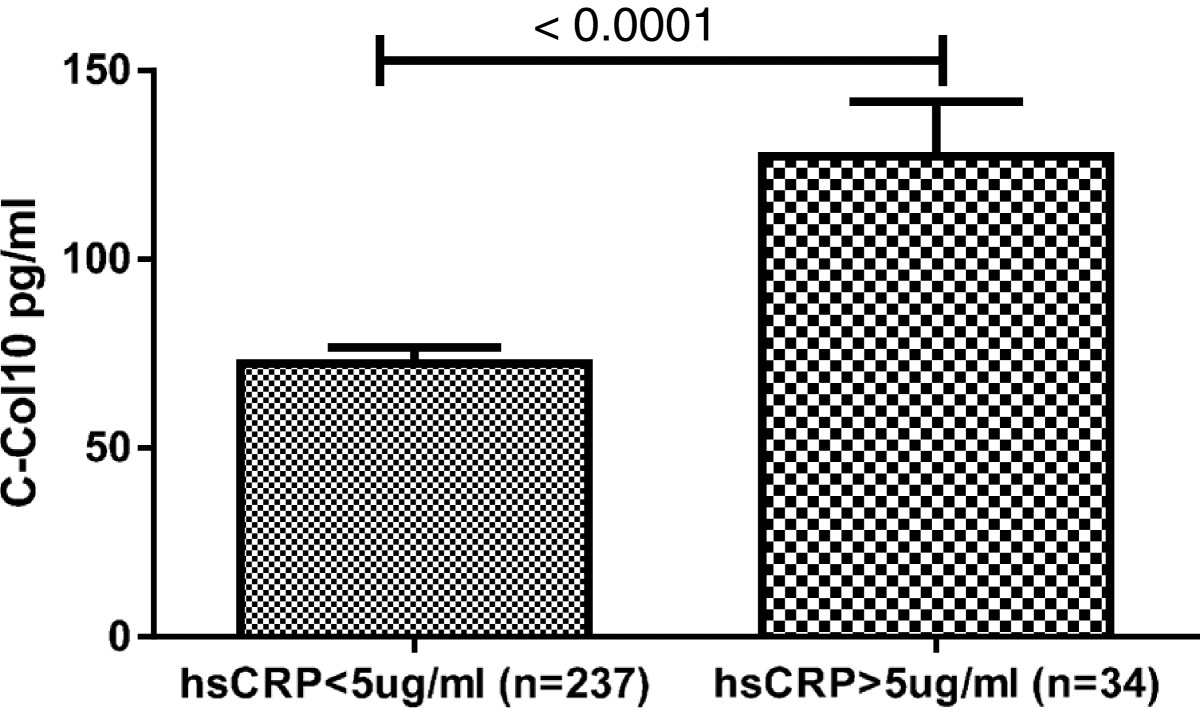
**Elevated C**-**Col10 levels in patients with above normal hsCRP levels,** **5 μg/** **ml.** T-test was used on log transformed data to determine the significant difference between these two groups.

271 subjects were divided into 5 groups based on their KL scores (Table 3). There was significant difference in the C-Col10 levels between KL0 and KL2 (p = 0.04). The mean value of C-Col10 in KL3 and KL 4 groups were 1.5 and 1.7 times higher but not statistically significant compared to KL0 group respectively, which could be high variation of individual C-Col10 levels in KL3 and KL4 groups. There was a trend towards elevated C2M level with increased KL score, but there was no significant difference existing between any two groups. The hsCRP levels did not show any correlation with the degree of KL score. There was a significant correlation between C-Col10 and hsCRP levels (r = 0.23, P < 0.0001), and between C-Col10 and C2M levels (r = 0.55, P <0.0001) (Table 4). No correlation between C2M and hsCRP has been found. Age and BMI adjustment did not change the significant correlations. OA patients with above normal hsCRP (>5) levels showed significantly increased C-Col10 levels (p < 0.0001) (Figure 4).

**Table 3:** **Serum levels of 3 biomarkers in 271 samples divided by KL score**

KL scroe	Number of Female/male	Age	BMI	C-Col10		hsCRP μg/ml	C2M pg/ml
				pg/ml	P value		
0	4/6	62.5 (57.3-67.7)	25.4 (23.7-27.0)	52 (24–80)	n. a.	2.94 (−0.75-6.64)	287 (245–325)
1	31/28	63.7 (61.6-65.8)	27.0 (26.0-28.1)	65 (54–76)	0.11	2.12 (1.44-2.80)	299 (270–327)
2	79/65	64.7 (63.5-65.9)	28.2 (27.5-28.8)	86 (73–98)	0.04*	3.18 (2.26-4.10)	302 (283–321)
3	17/19	64.3 (61.9-66.7)	29.3 (27.4-31.2)	80 (60–101)	0.07	3.57 (1.35-5.79)	305 (264–346)
4	12/10	67.8 (64.4-71.2)	29.5 (27.8-31.2)	87 (47–128)	0.28	2.92 (1.90-3.94)	339 (271–406)

The data is shown as mean [95-CI]. C-Col10: C-terminus of ColX assay; C2M: MMP-derived collagen type II fragment assay; hsCRP: high sensitive CRP assay. KL score: Kellgren-Lawrence score. One-way ANOVA was applied to compare the mean C-Col10 levels of each KL group with the mean of KL 0 group. P value was considered statistically significant if P < 0.05 and significant level was presented as: *.

**Table 4:** **Correlations between serum levels of the 3 biomarkers**

		C-Col10	C2M
**C**-**Col10**	Correlation Coefficient	n. a.	0.545
	Significance Level P	n. a.	<0.0001
**hsCRP**	Correlation Coefficient	0.233	0.071
	Significance Level P	<0.0001	0.2487

Pearson’s correlations were done on log transformed data. C-Col10: C-terminus of ColX assay; C2M: MMP-derived collagen type II fragment assay; hsCRP: high sensitive CRP assay.

### Immunolocalization of ColX in human OA cartilage by immunohistochemistry

**Figure 5:**
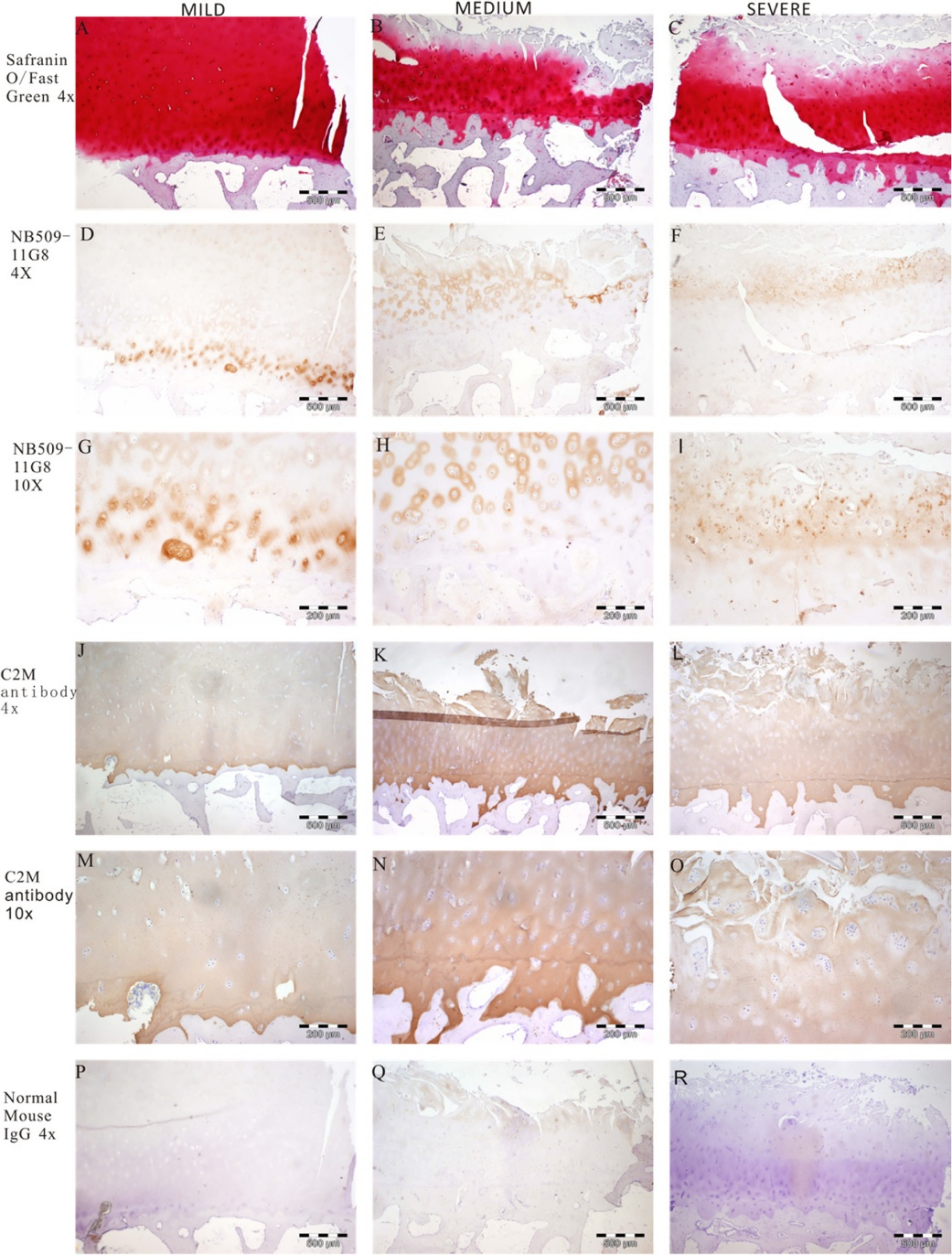
**Immunostaining of C**-**Col10 and C2M in human OA knee cartilage sections.** C**-** Col10 was observed in the deep zone around the pre**-** hypertrophic chondrocytes in mild OA, and around chondrocyte clusters in severe OA, whereas C2M was observed in all layers and consistently in 3 OA cartilage sections. **A**, **B**, **C**: Safranin O/ Fast green histology staining, 4× magnification, scale bar 500 μm; **D**, **E**, **F**: stained with NB509-11G8 antibody, 4× magnification, scale bar 500 μm; **G**, **H**, **I**: stained with NB509-11G8 antibody, 10x magnification, scale bar 200 μm. **J**, **K**, **L**: stained with C2M antibody, scale bar 500 μm; **M**, **N**, **O**: stained with C2M antibody, 10× magnification, scale bar 200 μm. **P**, **Q**, **R**: normal mouse IgG, negative control, scale bar 500 μm.

Cartilage biopsies including subchondral bone were stained with Safranin O/ Fast Green and classified according to severity of the cartilage damage (Figure 5A, B, C). There was a clear immune-reactivity of ColX in the deep zone and calcified cartilage matrix surrounding the hypertrophic chondrocytes in the mild OA section (Figure 5D, G). Staining of ColX was observed in the middle zone cartilage matrix in the moderate OA section (Figure 5E, H). The obvious staining of ColX was also observed in the matrix of clustered chondrocytes close to the fibrillated surface in the severe OA section (Figure 5F, I). C2M was observed in all layers and consistently in 3 specimens (Figure 5J-O). No obvious and specific staining was found in sections incubated with normal mouse IgG (negative control, Figure 5P-R).

## Discussion

In this study, we have developed and technically validated a novel and specific ELISA assay, C-Col10, which can determine the level of ColX in serum. We found significantly higher levels of ColX in patients with KL2 compared to patients with no radiographic sign of OA (KL0). Although the mean of ColX were markedly higher in KL3 and KL4 group than in KL0 group, these were not significantly different. We observed a high variation in the levels in these severe OA groups, which could indicate that different subgroups exist and ColX not alone was related to cartilage loss (hence joint space width) as observed by radiography. This was further supported by the significant correlation of between ColX and hsCRP and C2M levels, suggesting that chondrocyte hypertrophy was both associated with inflammation and cartilage degradation. In addition, immunostaining using the same monoclonal antibody as used in the C-Col10 assay revealed that ColX was predominately located around the hypertrophic chondrocytes located in the deep zone and around the clustered chondrocytes located in the middle and upper zone, indicating that C-Col10 measures were associated with differential changes of chondrocytes rather than solely degree of cartilage loss. To our knowledge, this is the first study to provide evidence of correlations between cartilage hypertrophy, inflammation and cartilage matrix degradation measured in OA serum samples.

A limitation of this study is the unavailability of recombinant human ColX protein for characterization of the antibody, NB509-11G8. Most commercial recombinant ColX proteins are fused with a partner at C-terminus, like 6-histidine tag. However, NB509-11G8 is a C-terminus specific antibody and the extra amino acids elongated at the C-terminus would influence the binding to NB509-11G8. Another limitation is the lack of detailed knowledge on the structure and the mechanism of release of ColX into circulation, thus there was no opportunity to make comparable judgment on the results that we have achieved. On the other hand our findings may add to the overall understanding of ColX in health and disease. Our findings identified a 17 kDa fragment in both the U2-OS cell lysate and human OA cartilage extract using the NB509-11G8 antibody, which align with the size of the fragment from C-terminus to potential cleavage site at Gly^479^-Ile^480^ bond, which has been identified in chick cartilage [13, 33]. In addition several other bands were identified indicating several structural entities of ColX, as well as several cleavage sites within the helical structure. The third limitation is that besides articular chondrocytes, subchondarl bone and osteophytes contribute to the serum measurement of ColX as well. We only did immunohistochemistry on human OA cartilage. We could image it will be quite interesting to investigate the presence and expression pattern of ColX in subchondral bone and osteophytes.

Several proteins have been associated with chondrocytes hypertrophy, among which expression analysis of *COL10A1* and *MMP13* are probably the most widely used markers of cartilage hypertrophy. However, synthesis of MMP13 can be induced not only by hypertrophic chondrocytes, but also by non-hypertrophic chondrocytes and other cells present in the joint through inflammation and mechanical stress [34, 35]. Type X collagen may potentially be a more specific marker of cartilage hypertrophy, as results on ColX seems to be limited to cartilage in the human adult. We have here presented data on the development and technically validation of a robust competitive assay, C-Col10. The level of C-Col10 was significantly correlated with level of C2M, a surrogate marker of cartilage degradation, which indicates a close relationship between chondrocytes hypertrophy and articular cartilage degradation. The direct link has been confirmed by the immunostaining of C-Col10 and C2M on cartilage biopsies taken from OA patients undergoing total knee replacement (TKR) surgery.

OA is normally considered as non-inflammatory joint disease, although it is evident that a subset of patients does indeed have synovitis (i.e. chronic inflammation). The hsCRP assay is used as standard diagnostic markers in inflammatory joint diseases such as RA; however the marker has limited usages in OA, since only a small proportion of the patients have elevated levels, although a few studies have shown hsCRP associated with clinical severity in patients with knee or hip OA [36, 37] and joint pain [38]. In our study, significantly higher C-Col10 levels were observed in patients with above normal hsCRP (>5 μg/ml) levels. This association between hsCRP and C-Col10 levels could indicate a causal relationship between inflammation and chondrocyte hypertrophy, which has been suggested through the activity of HIF-2α [39, 40]. The interpretation of this relation is consistent with previous studies that inflammatory factors, like interleukin-8 [41], Interleukin-1βinduced S100A11 protein [42] can accelerate chondrocyte hypertrophy.

There are contradictory data regarding hypertrophic chondrocyte in OA. Some publications have indicated that terminal differentiation of chondrocytes into hypertrophy is associated with OA [6, 43, 44]. Therefore hypertrophy chondrocyte is considered as one of the hallmarks in OA. In contrast, a study by Brew *et al*. demonstrated that hypertrophy associated genes expression, like collagen type X, was significantly down-regulated in OA cartilage [45]. The discrepancy has indicated the complexity and heterogeneity of the progression of OA disease, where different driving forces might be involved in. An earlier *ex vivo* study by Chen-An *et al*. demonstrated that the anti-resorptive drug, salmon calcitonin, was able to protect cartilage from hypertrophy in articular cartilage explants [46]. Our present study confirms and extends existing reports that the phenotype of chondrocytes changing into hypertrophy at least occurred in the subpopulation of patients with OA. Thus, anti-hypertrophic differentiation could be a critical factor considered for the future therapeutic OA drug development.

## Conclusions

In conclusion, chondrocyte hypertrophy and subsequent type X collagen seems to be increased in a subset of patients with inflammatory OA. The increase of ColX levels in serum possibly serve as a marker of local chondrocyte hypertrophy and differentiation, and may even aid in describing the progression of OA disease.

## Authors’ original submitted files for images

Below are the links to the authors’ original submitted files for images. Authors’ original file for figure 1 Authors’ original file for figure 2 Authors’ original file for figure 3 Authors’ original file for figure 4 Authors’ original file for figure 5

## Abbreviations

OA: Osteoarthritis
ELISA: Enzyme-Linked Immuno Sorbent Assay
KL score: Kellgren-Lawrence score
ColX: Collagen type X
hsCRP: high sensitivity CRP
C2M: MMP-derived collagen type II neoepitope.
