# Supplementary figures and images for: Type X collagen levels are elevated in serum from human osteoarthritis patients and associated with biomarkers of cartilage degradation and inflammation
Source: BMC Musculoskelet Disord. 2014 Sep 22;15:309. doi: 10.1186/1471-2474-15-309 (PMC4179849; doi:10.1186/1471-2474-15-309)

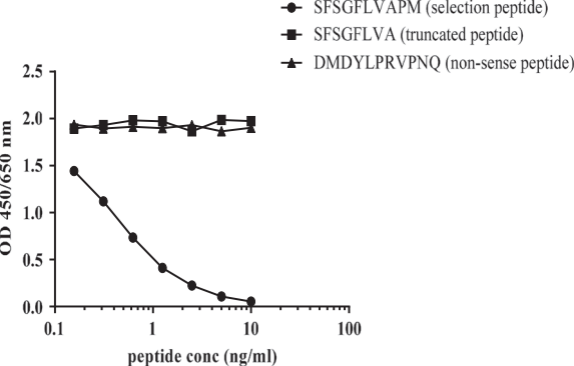

Supplement: Supplementary file 1 — Authors’ original file for figure 1 [file 12891_2013_2248_MOESM1_ESM.pdf]

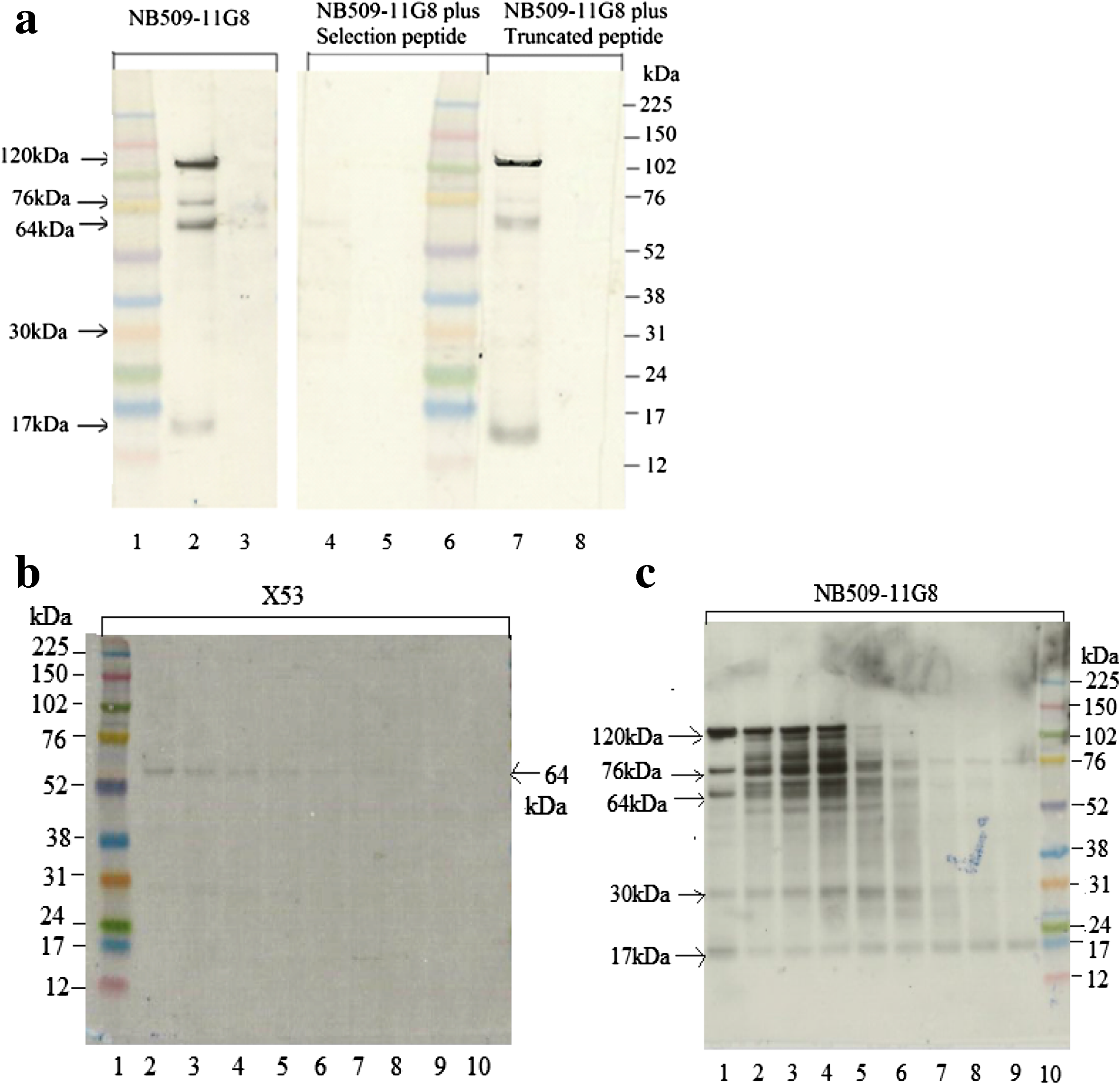

Supplement: Supplementary file 2 — Authors’ original file for figure 2 [file 12891_2013_2248_MOESM2_ESM.tif]

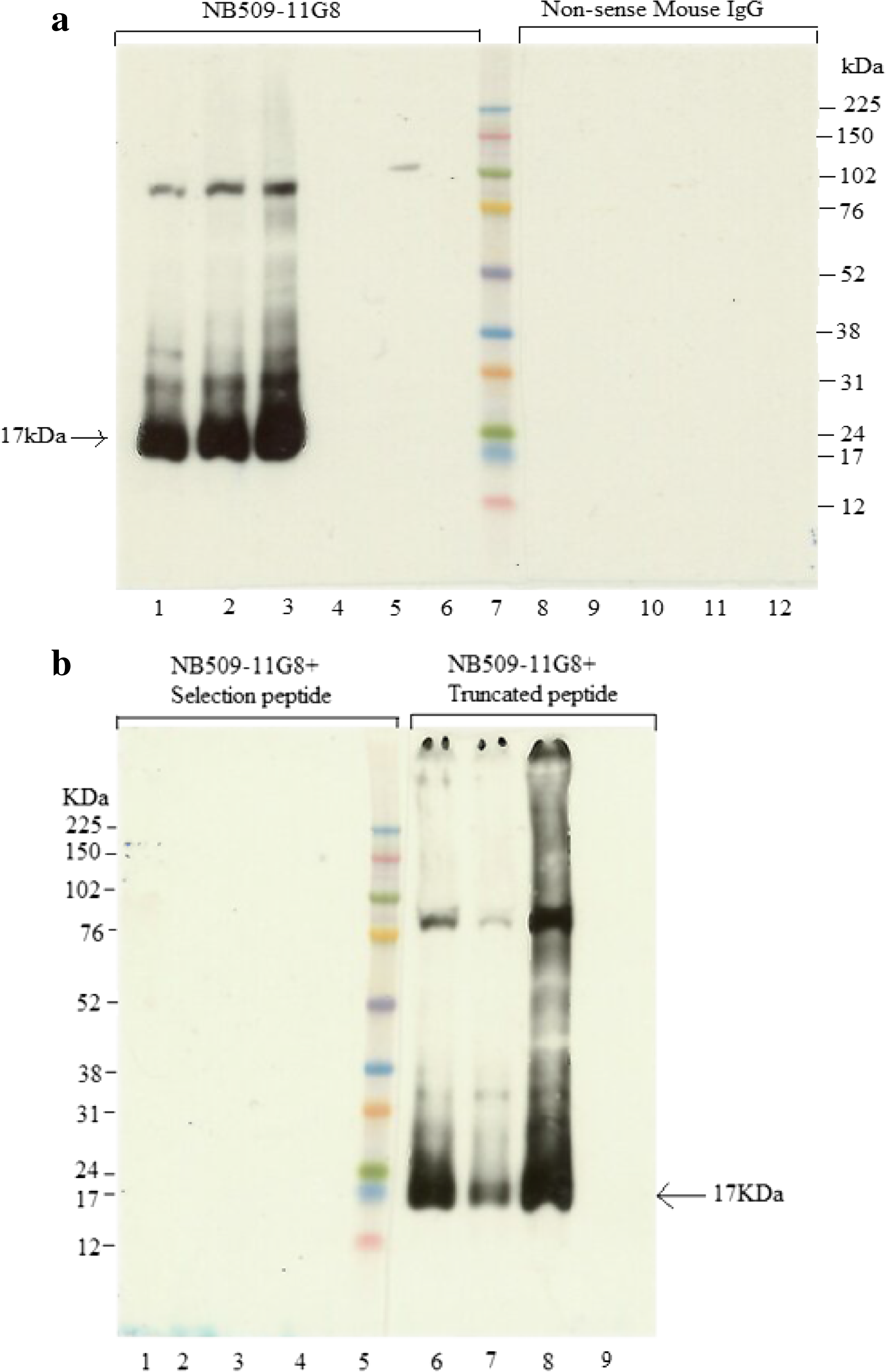

Supplement: Supplementary file 3 — Authors’ original file for figure 3 [file 12891_2013_2248_MOESM3_ESM.tif]

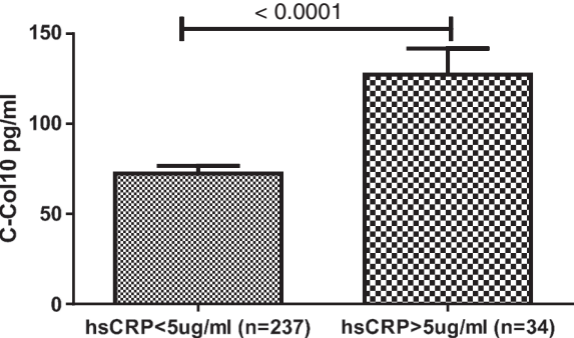

Supplement: Supplementary file 4 — Authors’ original file for figure 4 [file 12891_2013_2248_MOESM4_ESM.pdf]

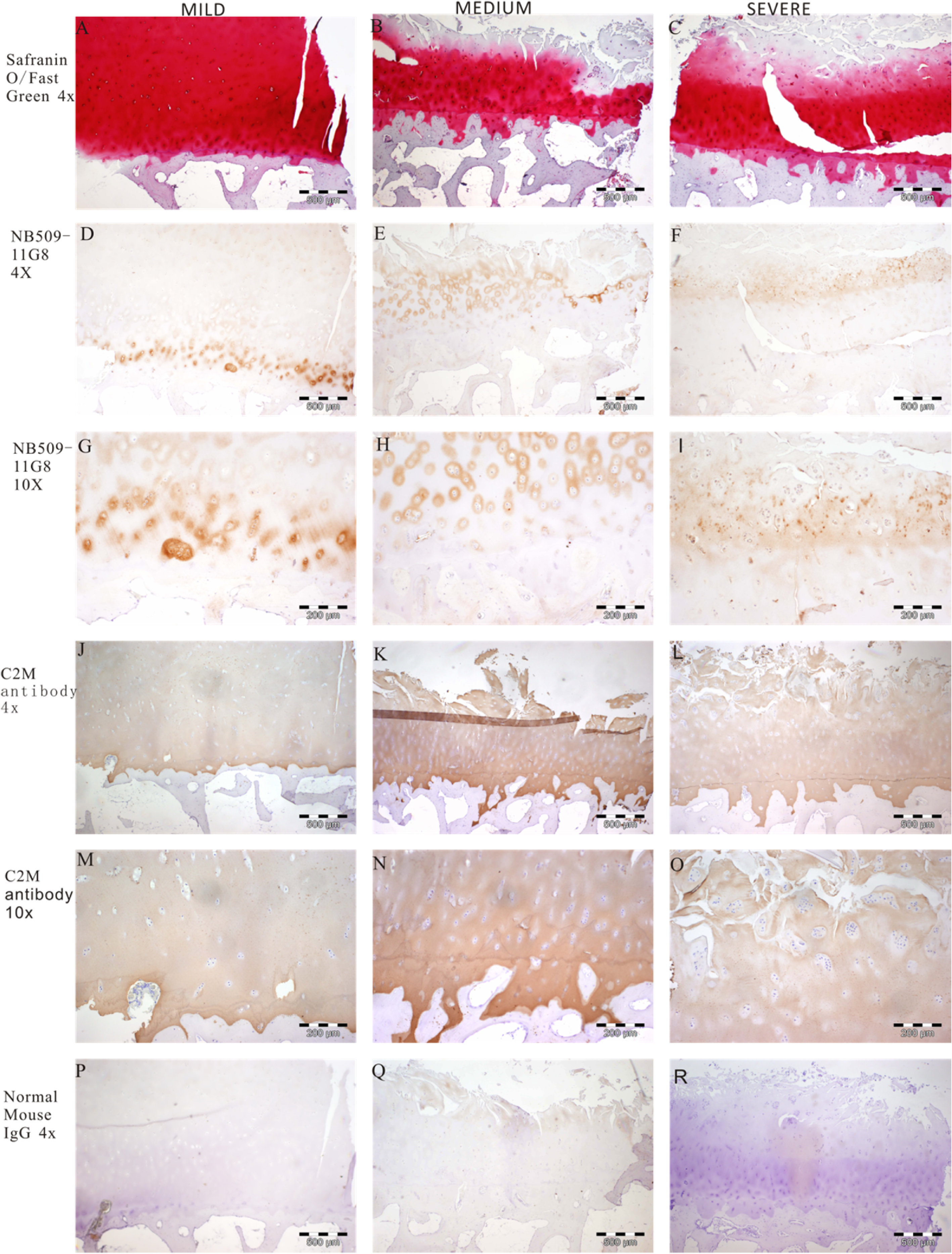

Supplement: Supplementary file 5 — Authors’ original file for figure 5 [file 12891_2013_2248_MOESM5_ESM.tif]
